# Echogenic Advantages of Ferrogels Filled with Magnetic Sub-Microparticles

**DOI:** 10.3390/bioengineering8100140

**Published:** 2021-10-11

**Authors:** Olga A. Dinislamova, Antonina V. Bugayova, Tatyana F. Shklyar, Alexander P. Safronov, Felix A. Blyakhman

**Affiliations:** 1 Department of Biomedical Physics and Engineering, Ural State Medical University, 620028 Ekaterinburg, Russia; o_dinislamova@rambler.ru (O.A.D.); bantonina1998@mail.ru (A.V.B.); t.f.shkliar@urfu.ru (T.F.S.); 2 Institute of Natural Sciences and Mathematics, Ural Federal University, 620002 Ekaterinburg, Russia; alexander.safronov@urfu.ru; 3 Institute of Electrophysics UB RAS, 620016 Ekaterinburg, Russia

**Keywords:** magnetic particles, ferrogels, medical ultrasound, sonography, bioengineering applications

## Abstract

Ultrasonic imaging of ferrogels (FGs) filled with magnetic nanoparticles does not reflect the inner structure of FGs due to the small size of particles. To determine whether larger particle size would improve the acoustic properties of FGs, biocompatible hydrogels filled with 100–400 nm iron oxide magnetic sub-microparticles with weight fraction up to 23.3% were synthesized and studied. Polymeric networks of synthesized FGs were comprised of chemically cross-linked polyacrylamide with interpenetrating physical network of natural polysaccharide—Guar or Xanthan. Cylindrical samples approximately 10 mm in height and 13 mm in diameter were immersed in a water bath and examined using medical ultrasound (8.5 MHz). The acoustic properties of FGs were characterized by the intensity of reflected echo signal. It was found that the echogenicity of sub-microparticles provides visualization not only of the outer geometry of the gel sample but of its inner structure as well. In particular, the echogenicity of FGs interior depended on the concentration of magnetic particles in the FGs network. The ultrasound monitoring of the shape, dimensions, and inner structure of FGs in the applied external magnetic field is demonstrated. It is especially valuable for the application of FGs in tissue engineering and regenerative medicine.

## 1. Introduction

The development of technologies for the synthesis of nano and sub-micro magnetic particles (MPs) has given a powerful impetus to the use of these materials in various fields of science and technology. In particular, biomedical applications of such particles are an intensively and dynamically developing area of bioengineering in the present days [1,2,3]. For instance, ferrofluids with MPs monitored and controlled by combination of high-tech physical methods are introduced for the diagnosis and treatment of diseases, e.g., cancerous tumors [4,5,6].

Magnetic particles can also be used as a filler for biocompatible synthetic composites which are sensitive to an external magnetic field. From the viewpoint of bioengineering applications, such systems are mainly introduced as magnetoactive actuators, artificial muscles or valves, etc. [7,8].

Among the variety of magnetic composite materials filled with MPs, the hydrogel-based magnetic composites are in the focus of bioengineering applications. A hydrogel is a polymeric network swollen in water [9,10,11]. Hydrogels are able to contain more than 99% of water by volume, which makes them compositionally similar to biological tissues. Modern synthetic approaches make it possible to introduce biomacromolecules and nano- and sub-micro sized MPs into polymeric networks of hydrogels. Ferrogels (FGs) with embedded magnetic particles are promising materials for the needs of biosensing, cellular technologies and regenerative medicine, in particular [12,13,14,15,16,17,18,19,20].

Two-dimensional and three-dimensional matrixes (scaffolds) for cell cultivation based on FGs have a number of advantages over other magnetic composites. They have good biocompatibility, variability of elastic properties specified during the synthesis [21,22,23], and provide the ability to control these properties using an external magnetic field [24,25,26]. Such features offer additional opportunities for the optimization of adhesion, proliferation, and differentiation of cells on scaffolds. It might especially be valuable for the use of FGs as implants for tissue replacement therapy [27,28,29,30].

The use of FGs as implants in vivo requires their reliable visualization. Potentially, medical ultrasound, which is the most affordable and the safest method of medical diagnostics, provides such an opportunity. Under certain conditions the ultrasound diagnostics can be used to obtain quantitative information on the size and/or concentration of particles in suspensions [31,32,33,34,35] or on the microstructural parameters of inhomogeneous media [36]. A number of studies had demonstrated the advantages of ultrasonic techniques for detection of various kinds of particles in fluids and soft materials [37,38], including gels and ferrogels [39,40].

In our recent study, we had studied the echogenicity of FGs based on polyacrylamide filled with magnetic iron oxide nanoparticles [40]. It was found that in such composites the boundary FG/water was clearly visualized by ultrasonic detection. Moreover, the intensity of the echo signal reflected from the surface of FG in water depended on the concentration of nanoparticles in the ferrogel. This effect correlated with the influence of nanoparticles on the FGs elastic properties, in particular, with the increase in Young’s modulus of FG with increasing concentration of MPs. At the same time, the reflection of ultrasound was observed only at the FG boundary. The interior of FG did not show any echogenicity if subjected to medical ultrasound (frequency of 8.5 MHz), which obviously was due to the small particle size (14 nm in diameter).

Meanwhile, the ultrasound monitoring of FGs interior is necessary if they are subjected to the external magnetic field which affects both the geometry and the internal structure [41,42]. Therefore, the complex ultrasonic detection of FGs may provide useful information on the cell proliferation rate, the stages of tissue regeneration, the degradation of implants, and so on [21,43,44].

The present study was focused on the features of ultrasound imaging of FGs filled with iron oxide sub-microparticles in a wide range of MPs content. A biocompatible hydrogel with interpenetrating polymer networks—chemical, based on polyacrylamide, and physical, based on natural polysaccharides (Guar or Xanthan)—was used as a matrix for FGs. It was shown that the inclusion of 100–400 nm iron oxide sub-microparticles in the gel opened up the possibility of visualizing the interior of ferrogels using standard medical ultrasound instruments. The echogenicity of the interior of the ferrogel was found to depend on the concentration of MPs in the FG network.

## 2. Materials and Methods

### 2.1. Synthesis of Ferrogels

**Figure 1:**
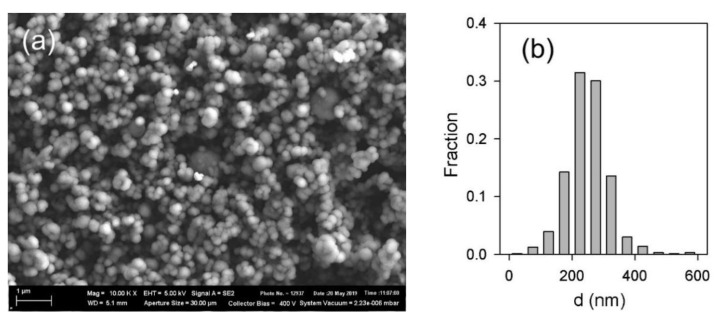
(**a**) SEM image of magnetite particles; (**b**) particle size distribution given by graphical image analysis of 632 particles in spherical approximation.

Commercial iron oxide: magnetite Fe_3_O_4_ (Alfa Aesar, Ward Hill, MA, USA), was used as a magnetic filler for the synthesis of ferrogels (FGs). SEM image (Carl-Zeiss Auriga Cross-beam, Zeiss, Oberkochen, Germany) of magnetite particles is presented in Figure 1. The particles were quasi-spherical with caliper diameter 100–400 nm. Graphical analysis of 632 images gave the particle size distribution (PSD) histogram which is shown in the inset in Figure 1. The median of the distribution was 250 nm, calculated weight average caliper diameter was 305 nm.

The specific surface area of iron oxide MPs was measured via low temperature sorption of nitrogen (Micromeritics TriStar3000, Micromeritics Instrument Corp., Norcross, GA, USA) and was found *S* = 6.9 m^2^/g using Brunauer–Emmett–Teller (BET) approximation. Using this value, the apparent particle diameter (*d_BET_*) was calculated in spherical approximation using the following equation:

(1) $$d_{BET}=\frac{6000}{\rho *S}$$

Taking the crystallographic density of magnetite ρ = 4.6 g/cm^3^ the apparent diameter was *d_BET_* = 190 nm. This value is lower than that evaluated from PSD histogram likely due to the deviation of particle shapes from sphericity. 

Phase composition of iron oxide was characterized using XRD (Bruker D8 Discover, Billerica, MA, USA). It contained 94% of magnetite, 5% of goethite, and 1% of hematite. XRD diffractogram is given in the Supplementary Information (Figure S1).

Magnetic properties were characterized by vibrating sample magnetometry (Cryogenics, Ltd. VSM, London, UK). According to magnetic hysteresis loop saturation magnetization of iron oxide particles was 84 emu/g, remanence was 6.6 emu/g, and coercitivity was 78 Oe. Magnetic hysteresis loop is given in Supplementary Information (Figure S2).

Synthesis of ferrogels was carried out by free radical polymerization of acrylamide (AAm) monomer in water solution. Prior to the synthesis water solutions of other reagents were prepared. Namely: 48 mM solution of a cross-linker N,N′-methylenediacrylamide (MDAA), 50 mM solution of an initiator ammonium persulfate (PSA), and 1% (wt.) solutions of polysaccharide thickeners Guar and Xanthan. If sub-micron magnetic particles are to be embedded in ferrogels there is a problem of the sedimentation of particles in the reaction mixture during polymerization in AAm water solution. A way to overcome it is the elevation of the viscosity of the reaction mixture, which prevents sedimentation of sub-micron solid particles during the synthesis. The most efficient thickeners to raise the viscosity are natural polysaccharides, which provide physical gelation in their water solutions in low concentration and slow down sedimentation of embedded particles.

The weighted portion of iron oxide was vigorously mixed with water (0.5 mL) and MDAA solution (0.75 mL). Then suspension was homogenized for 25 min in ultrasound bath. AAm was dissolved in this suspension and 2.5 mL of thickener (Guar or Xanthan) solution was added. After complete dissolution and homogenization solution of PSA (0.5 mL) was added and the reaction mixture was placed into cylindrical polyethylene tube (8 mm in diameter). Total volume of the mixture was 4.5 mL, the concentration of AAm monomer was 0.8 M, and MDAA to AAm ratio was 1:100. Polymerization took place for 1 h at 80 °C. Two series of ferrogels were synthesized: one with Guar the other with Xanthan. In both series the content of iron oxide in reaction mixture varied from 0 to 30% (wt.)

After the synthesis ferrogels were taken out of the molds and were washed in distilled water for 2 weeks with daily water renewal to remove salts, unreacted monomers, and linear PAAm oligomers. Swelling ratio of ferrogels was monitored until it reached the constant equilibrium level. The final content of iron oxide in swollen FGs was determined by thermogravimetric analysis using thermal analyzer NETSCH STA409 (NETZSCH Geratebau, Selb/Bavaria, Germany). First, ferrogel samples were dried to the constant weight at 70 °C to determine water content. Then, dry residues were heated from 40 up to 1000 °C at 10 K/min in the air flow 20 mL/min. The organic phase of ferrogel decomposed. Then, the weight loss gave the polymer content, and the weight of the residue gave the iron oxide content. FGs based on PAAm/Xanthan network contained 0.0%, 0.6%, 1.9%, 4.3%, 5.7%, 8.2%, 12.9%, 22.3% of iron oxide MPs by weight. FGs with PAAm/Guar network contained 0.0%, 0.1%, 2.9%, 4.6%, 10.3%, 16.0%, 23.3% of iron oxide MPs by weight.

**Figure 2:**
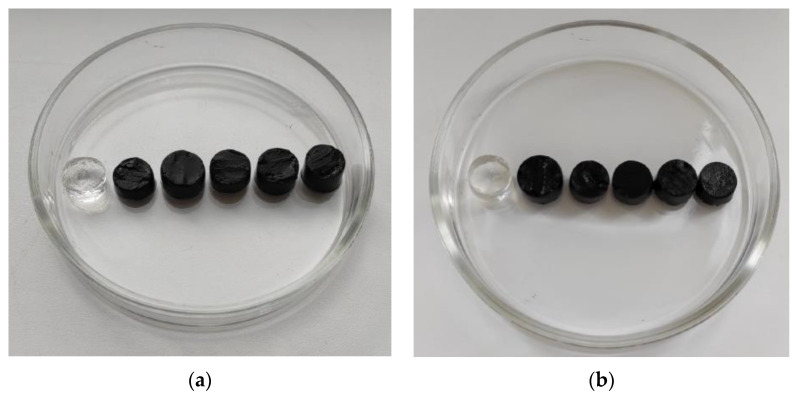
General view of PAAm/Xanthan (**a**) and PAAm/Guar (**b**) FGs samples filled with MPs at various weight fractions. (**a**) Weight fraction of MPs: 0.0%, 1.9%, 4.3%, 5.7%, 12.9%, 22.3%; (**b**) 0.0%, 2.9%, 4.6%, 10.3%, 16.0%, 23.3% (from the left to the right). Diameter of samples is about 13 mm.

After the equilibration ferrogels were cut into cylinders approximately 10 mm in height and 12–14 mm in diameter. The general view of FG samples is given in Figure 2. 

### 2.2. Ferrogel Echogenicity Measurements

**Figure 3:**
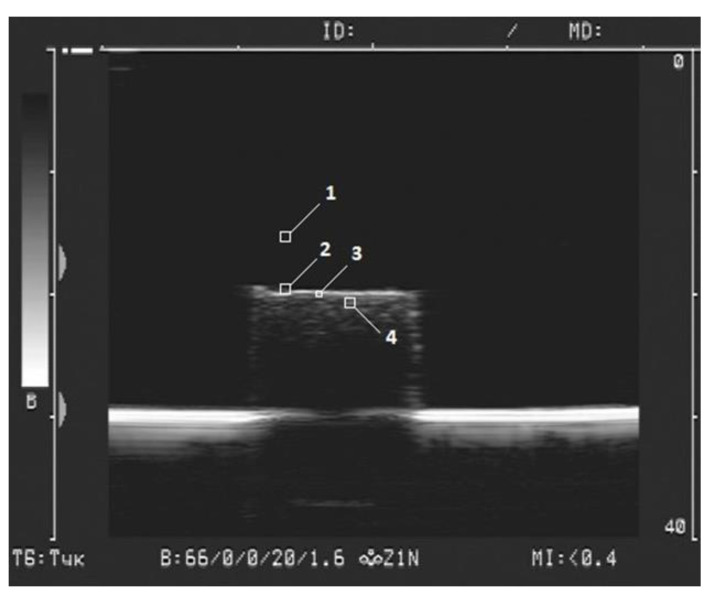
Example of ferrogel visualization. (PAAm/Xanthan, 12.9% of MPs). Squares with marks 1–4 illustrate locations where specific parameters of brightness were calculated. See also explanation in the text.

The details of visualization of FGs were described in our earlier study [40]. Briefly, a Sonoline Adara (Siemens, Munich, Germany) medical instrument with a SIEMENS 7.5L45s Prima/Adara linear sensor was used. The following hardware settings were applied: the dynamic range of the ultrasonic device—66 dB, the operating frequency—8.5 MHz, the wavelength—0.17 mm, the gain—20 dB, and the power—1.6%. Samples of FGs were placed at the bottom of a cuvette filled with 500 mL of distilled water. The sensor was immersed in water and installed at a height of 30 mm above the surface of sample. The image of samples in two-dimensional (2D) mode in gray scale was recorded in a video file with a duration of several seconds with a frame rate of 25 frames per second, and the frame size was 720 pixels × 576 pixels. An example of ferrogel visualization is shown in Figure 3. 

Special software was developed and used to quantify the brightness of different areas of the image. It allowed to measure the brightness in the vicinity of a point specified by the user. The brightness was characterized in arbitrary units and it ranged from 0 (black) to 255 (white).

To determine the maximal brightness parameter the boundary line of the gel was split into squares 10 pixels × 10 pixels in each (Figure 3, mark 2). Linear dimensions of the square were 0.7 mm × 0.7 mm. For each FG sample the number of squares at the boundary was 12–15. The brightest pixel in each square was recorded. The number of samples with the same composition was 5, thus the total number of maximal brightness values was 60–75 for each composition of FG. The average value was taken for the analysis.

To determine the average brightness of the gel surface the boundary was split into squares 3 pixels × 3 pixels in each (Figure 3, mark 3). Linear dimensions were 0.2 mm × 0.2 mm. In each square the average brightness of pixels was calculated. The values for all squares at the boundary were then averaged over all the squares and the average brightness was taken for further analysis.

The maximal and the average brightness were also evaluated in the interior of FG (Figure 3, mark 4). Therefore, the described splitting procedure was performed beneath the boundary of the gel approximately 2 mm below its surface. 

For each type of FG, the average value of the maximum and average brightness, as well as the limits of the confidence interval at a significance level of *p* = 0.01, were calculated. In all experiments, 10 pixels × 10 pixels square image was used to estimate the background (Figure 3, mark 1). The average value of the maximum brightness of the water in the cuvette in all tests of the gels was 33.1 ± 0.2 (*n* = 70).

### 2.3. Measurement of Ferrogel Viscoelasticity

A laboratory setup for mechanical tests was used to evaluate FGs viscoelasticity. The same methodical approach was used in a number of our earlier studies [17,22]. Briefly, FGs cylindrical samples were placed between two plates. One was connected rigidly to an actuator of a linear electromagnetic motor, and the other was connected to a precision force transducer. The motor induced compression strain with dynamic (sine-wave) mode with a magnitude of up to 15% of the initial gel length. The elastic storage modulus (G’) and the loss modulus (G’’) were calculated at a frequency range of 0.05 Hz.

## 3. Results

### 3.1. Characterization of Ferrogels

**Figure 4:**
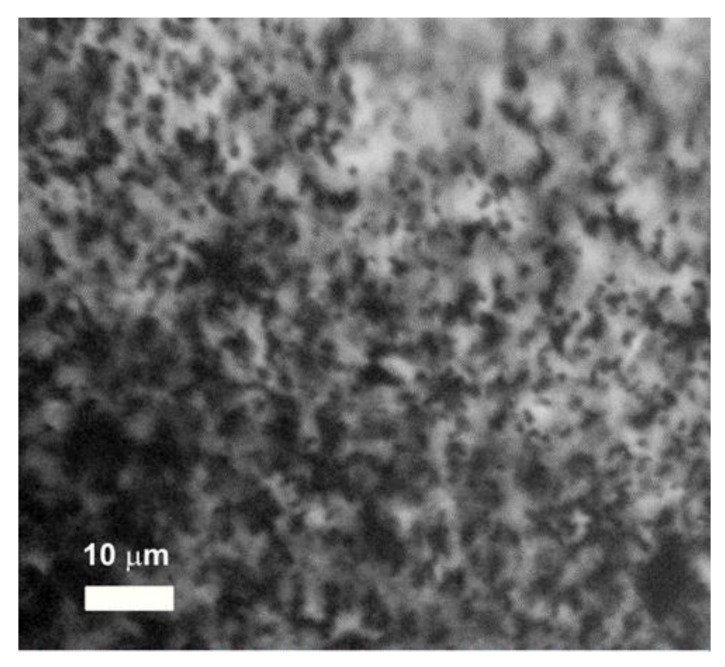
Optical microphotograph of FG with PAAm/Xanthan network and iron oxide content 1.9% (wt.). Image obtained by “Micromed-I” microscope (Micromed Ltd., St. Petersburg, Russia).

Figure 4 presents an optical microphotograph of FG with PAAm/Xanthan network filled with 1.9% of iron oxide. It could be noticed that the distribution of MPs in gel matrix was uniform. There were no large aggregates, and the caliper diameter of the observed inclusions was less than 1 µm. Most likely, the dark spots in Figure 4 correspond either to individual iron oxide MPs or to small aggregates which combine few particles only.

**Figure 5:**
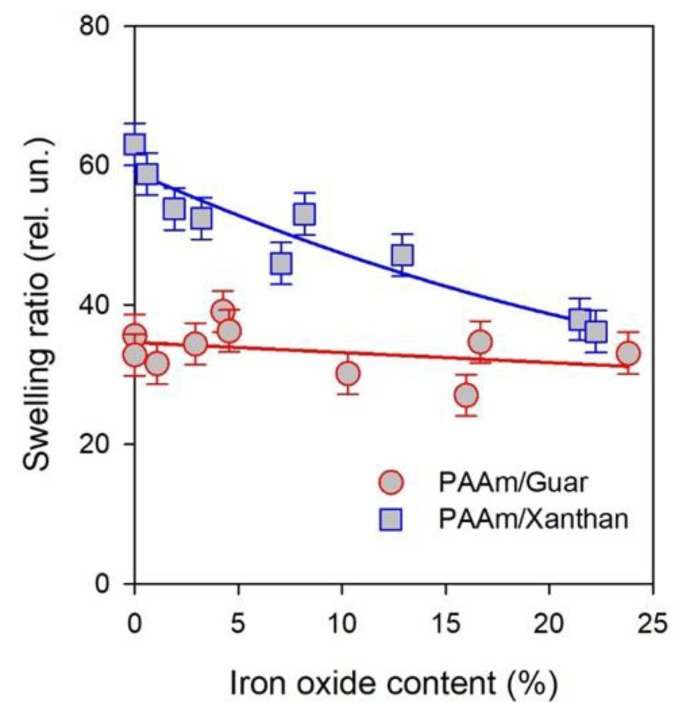
Dependence of swelling ratio of FG in PAAm/Guar and PAAm/Xanthan series on the content of iron oxide MPs. Lines are given for an eye-guide only.

The basic feature of gel is its swelling ratio, which is the water uptake by the unit mass of dry polymeric network. Swelling ratio depends on a number of factors of inner gel structure. For instance, it depends on the networking density, on the molecular interactions between polymer chains and water molecules, on the presence of other polymers and solid particles, etc. Figure 5 presents dependence of the swelling ratio on the iron oxide content in FGs of two series. Note that presented values of the swelling ratio correspond to the polymeric phase only excluding the fraction of solid MPs.

The swelling ratio in FGs with PAAm/Guar network was almost independent on iron oxide content. The swelling ratio of a blank PAAm/Guar hydrogel was around 35. With the embedding of iron oxide MPs, a little decrease might be noticed. However, it might rather be within the limits of experimental error for swelling ratio.

The trend of swelling ratio in FGs with PAAm/Xanthan network is more pronounced. First, the blank PAAm/Xanthan hydrogel contained almost two times larger amount of water than PAAm/Guar hydrogel. Swelling ratio of PAAm/Xanthan hydrogel was around 60. Both hydrogels had the same overall concentration of AAm in the synthesis and the same cross-linking density of the chemical networking. The only difference was the nature of polysaccharide thickener. Polysaccharides are known to form physical gels in their water solutions due to the cross-linking by interchain hydrogen bonds. Therefore, the structure of PAAm/Guar and PAAm/Xanthan hydrogels consists of two interpenetrating networks. One of which is a chemical one provided by copolymerization of AAm with DMAA, the other is a physical network of polysaccharide. The difference in swelling ratio between PAAm/Guar and PAAm/Xanthan blank hydrogels indicated that the double networks in these two cases were different. Supposedly, it might be the result of different conformations of Guar or Xanthan macromolecules in the interpenetrating chemical and physical networks of gel. There might also be differences in the molecular interaction between Guar or Xanthan with the PAAm network. For now, the reasons for the difference between Guar or Xanthan influence on the swelling of PAAm gel are unclear.

The influence of iron oxide content of the swelling ratio of PAAm/Xanthan FGs was a diminishing trend. At MPs content around 20% the swelling ratio was 2/3 of the swelling ratio of the blank gel. At high load of iron oxide FGs with the PAAm/Xanthan network had almost the same water uptake as FGs with PAAm/Guar network. As the underlying reasons for the difference in the swelling of PAAm/Xanthan or PAAm/Guar blank gels are obscure, it is difficult to rationalize the influence of the iron oxide solid particles. The only conclusion that might be made is that the presence of solid particles somehow compensates the difference of the polysaccharides in the double interpenetrating network.

### 3.2. Effect of MPs Concentration on FGs Viscoelastic Properties

**Figure 6:**
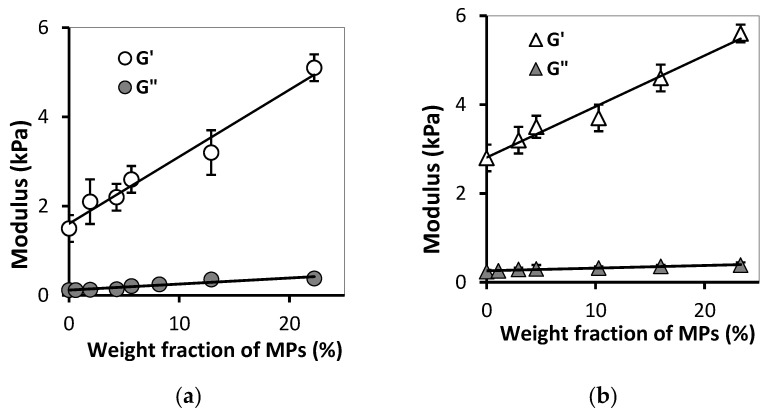
Dependences of storage modulus (G’) and loss modulus (G’’) on the MPs concentration for samples PAAm/Xanthan (**a**) and PAAm/Guar (**b**). Vertical bars reflect the confidence interval with *p* = 0.01 (*n* = 5). Equations of linear regression are: (**a**) y = 0.15x + 1.60 (R^2^ = 0.973) for G’ and y = 0.01x + 0.11 (R^2^ = 0.85) for G’’; (**b**) y = 0.11x + 2.81 (R^2^ = 0.98) for G’ and y = 0.01x + 0.26 (R^2^ = 0.95) for G’’.

Figure 6 shows the dependence of the mechanical characteristics of gels on the concentration of MPs for two series of ferrogels. One can see that an increase in the MPs concentration resulted in gradual increase in both the storage modulus and the loss modulus. Meanwhile, this relationship for the storage modulus was strong, while for the loss modulus the differences in values were within the experimental error. At any given concentration of MPs, PAAm/Guar and PAAm/Xanthan FGs gels demonstrated the dominance of elastic behavior: the storage modulus was several times larger than the loss modulus. In both series of FGs the dependence of the storage modulus on the content of MPs was close to linear. 

Thus, the addition of iron oxide MPs to the gel network resulted in the increase of elasticity in composites based on PAAm and polysaccharides. Moreover, the effect of MPs concentration on the FGs viscoelastic properties was approximately the same in two tested series of ferrogels.

### 3.3. Effect of MPs Concentration on FGs Echogenicity

**Figure 7:**
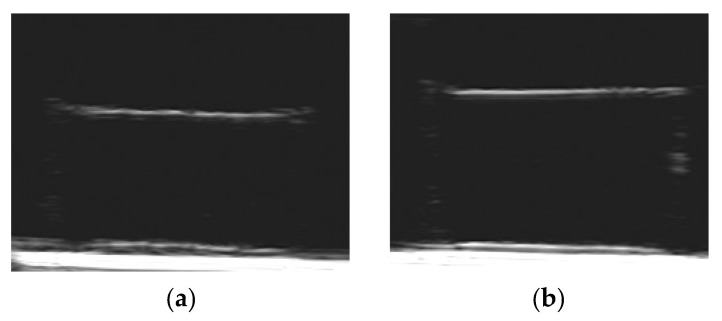

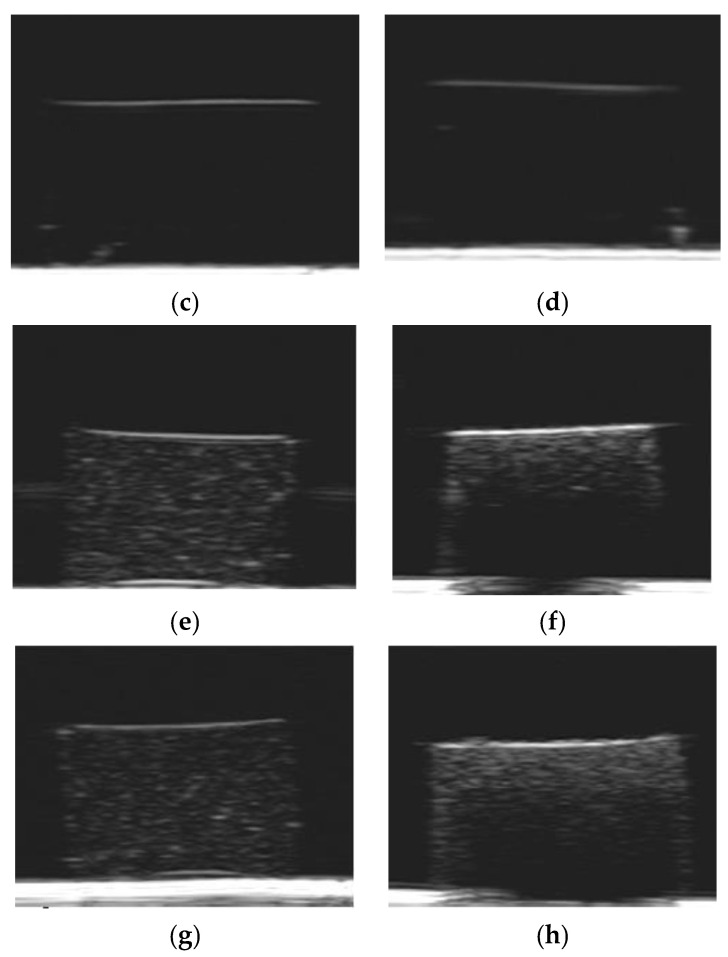
Examples of ultrasound imaging of gels with various content of fillers. (**a**) PAAm gel; (**b**) PAAm gel filled with iron oxide nanoparticles (~14 nm in diameter), weight fraction of 2%; (**c**) PAAm/Xanthan blank gel; (**d**) PAAm/Guar blank gel; (**e**) PAAm/Xanthan gel filled with iron oxide microparticles, weight fraction 1.9%; (**f**) PAAm/Xanthan gel filled with iron oxide microparticles, weight fraction 22.3%; (**g**) PAAm/Guar gel filled with iron oxide microparticles, weight fraction 2.9%; (**h**) PAAm/Guar gel filled with iron oxide microparticles, weight fraction 23.3%.

Figure 7 demonstrates some examples of FGs visualization obtained in both the series of ferrogels filled with iron oxide MPs (e–h). The ultrasound images for blank PAAm gel (a), for PAAm ferrogel filled with iron oxide magnetic nanoparticles (b), for blank PAAm/Xanthan gel (c) and for blank PAAm/Guar gel (d) are also presented for comparison. One can see that the boundary of gel/water was clearly visualized in all presented samples. At the same time, the echogenicity inside the blank PAAm gel (a), ferrogel with magnetic nanoparticles (b) and blank gels with interpenetrating polymer networks (c,d) did not differ from the background (water).

On the contrary, the FGs under the present study (e–h) demonstrated additionally the reflected echo signal from the interior of samples. Visually, the brightness inside the sample enlarged higher was the MPs concentration. It implies that the intensity of the reflected echo signal depended on the MPs concentration in both series of composites: FGs PAAm/Xanthan (e,f) and FGs PAAm/Guar (g,h). Furthermore, in FGs filled with the highest concentration of MPs (f,h) the brightness intensity inside the sample decreased with the increase of the distance from the upper boundary of the composite.

**Figure 8:**
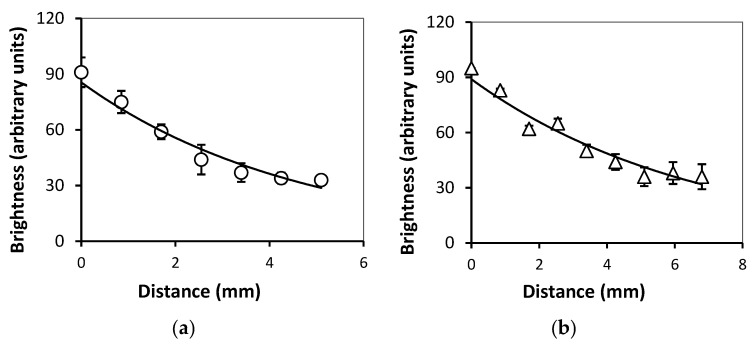
Dependences of maximal brightness inside the FGs on the distance from the upper gel/water boundary in PAAm/Xanthan sample (**a**) and PAAm/Guar sample (**b**). Vertical bars reflect the confidence interval with *p* = 0.01 (*n* = 13). See also explanation in the text.

Figure 8 presents the dependence of maximal brightest of the echo signal on the distance from below the boundary of FGs (see Figure 7f,h). One can see that the intensity of reflected echo signal (*I*) inside the FGs diminishes exponentially with the increase in the distance from the gel/water boundary. The exponential decay function for the case of FG PAAm/Xanthan with 22.3% of iron oxide was *I* = 92.7e^−d/4.7^ (R^2^ = 0.94). For FG PAAm/Guar with 23.3% of MPs it was *I* = 89.6e^−d/6.6^ (R^2^ = 0.91). It means that the characteristic distance for the damping of reflected echo signal in ferrogels with iron oxide MPs with average diameter 300 nm was around 5 mm.

Table 1 presents the results of the statistical analysis of the ultrasound visualization for ferrogels with the PAAm/Xanthan network with different content of embedded iron oxide MPs. Table 2 provides the same information for FGs with the PAAm/Guar network. In general, the increase of MPs content resulted in the enhancement of all echogenic parameters in both series of ferrogels. The maximal brightness at the boundary and in the interior was reliably larger than the average values. The increase in the load of MPs was also accompanied with the enlargement of the standard deviation of the values of brightness. Most likely, it reflected the increase of the structural inhomogeneity inside FGs. 

**Figure 9:**
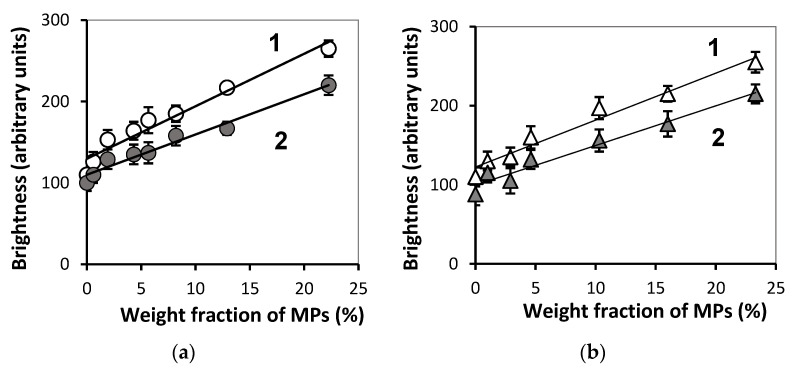
Dependences of maximal (1) and average brightness (2) at gel/water boundary on the MPs concentration for samples PAAm/Xanthan (**a**) and PAAm/Guar (**b**). Vertical bars reflect the confidence interval with *p* = 0.01 (*n* = 60). Linear regressions with *x* being the concentration of iron oxide MPs in % (wt.): (**a**) *I_max_* = 6.5*x* + 129.6 (R^2^ = 0.953), *I_av_* = 4.9*x* + 110 (R^2^ = 0.968); (**b**) *I_max_* = 5.9*x* + 122.4 (R^2^ = 0.969), *I_av_* = 5.0*x* + 99.5 (R^2^ = 0.962).

Figure 9 gives the dependence of the maximal and the average brightness at the boundary of FGs in two series. The increase in MPs concentration provided the linear enhancement of the reflected echo signal from the gel/water boundary. Corresponding linear regressions for the maximal (*I_max_*) and the average (*I_av_*) brightness are presented in the figure caption.

**Figure 10:**
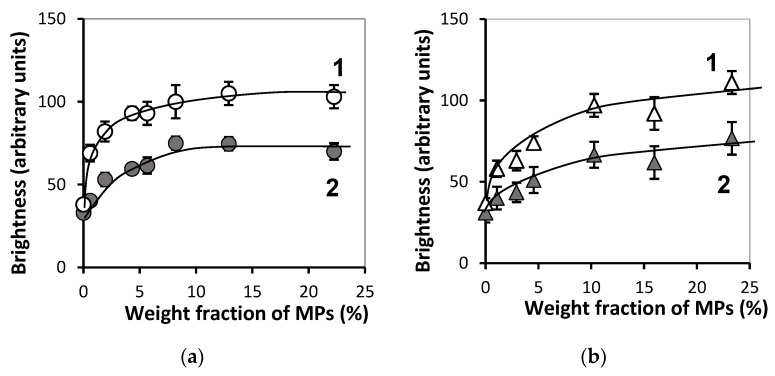
Dependences of maximal (1) and average brightness (2) at gel bulk on the MPs concentration for samples PAAm/Xanthan (**a**) and PAAm/Guar (**b**). Vertical bars reflect the confidence interval with *p* = 0.01.

Figure 10 presents concentration dependences for the maximal and average brightness of the interior of FGs in both series. It is noticeable that the addition of the first portions of MPs substantially enhanced the brightness of the reflected echo signal. Meanwhile, with further increase in MPs content the dependence tended to saturation unlike the linear growth in the case of the brightness at the boundary. 

**Figure 11:**
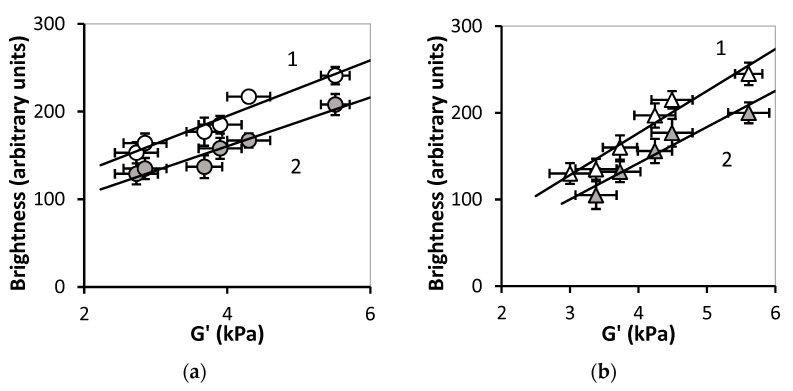
Dependences of the maximum (1) and average (2) brightness on the storage modulus for PAAm/Xanthan (**a**) and PAAm/Guar (**b**). Linear regressions: (**a**) *I_max_* = 31.7*G*’ + 68.1 (R^2^ = 0.946), *I_av_* = 27.8*G*’ + 49.2 (R^2^ = 0.930); (**b**) *I_max_* = 48.5*G*’ − 17.1 (R^2^ = 0.950), *I_av_* = 41.7*G*’ − 25 (R^2^ = 0.921).

Figure 11 shows the correlation trends between the maximal and average brightness of the reflected echo signal on one side and the storage modulus of FGs on the other. Correlations fairly well match the linear regressions.

## 4. Discussion

The specific features of the ultrasound visualization of ferrogels filled with iron oxide magnetic sub-microparticles with an average diameter 300 nm were studied in the present work. Polyacrylamide hydrogel was used as a basic platform for these FGs. Additionally, the polymeric matrix of ferrogels included natural polysaccharides—Xanthan or Guar. 

The advantages of gels and ferrogels with interpenetrating network for applications in the biomedical engineering were proven in a number of studies [45,46,47,48,49,50]. Furthermore, the use of magnetic microparticles in comparison with nanoparticles is considered more justified for biomedicine from the viewpoint of the material toxicity [51,52,53].

It is noteworthy that the acoustic impedance of the gels under study and some biological tissues is close. In this work, a limited number of samples from two series of FGs were tested by the echo-pulse method using an “Introtest-1MV” flaw detector (Introtest Ltd., Yekaterinburg, RF). In gels filled with MPs weigh fraction from 0% to 3%, the acoustic impedance varied from 1.3 to 1.4 × 10^6^ Pa·s/m, which corresponds to the acoustic impedance of adipose tissue. For ferrogels with a particle concentration of more than 4%, the acoustic impedance ranged from 1.6 to 2.1 × 10^6^ Pa·s/m, which corresponds to the acoustic impedance of muscle tissue [54].

Meanwhile, the FGs filled with sub-microparticles demonstrated acoustic reflection not only from the gel/water boundary but from the interior as well. In this respect, FGs with embedded iron oxide sub-microparticles are different from ferrogels with iron oxide nanoparticles with an average diameter 10–20 nm [40]. In the latter case the echo signal from the interior of ferrogel was the same as for water itself. The illustration is given in Figure 7a,b.

Based on the straightforward physical evaluation, the individual solid particles with a caliper diameter 100–400 nm cannot be visualized with the medical ultrasound instruments, which usually operate at 8.5 MHz frequency. Given the sound wave velocity in water 1500 m/s the evaluation gives 176 µm for the wavelength of the ultrasound of the commercial medical instruments. Thus, the space resolution of ultrasound instruments is three orders of magnitude coarser than the characteristic dimensions of particles. Of course, the aggregation of particles can diminish the gap between ultrasound wavelength and the characteristic dimensions of visualized inclusions. However, as shown in Figure 4 the aggregates in FGs did not exceed several microns, and it was still much lower that the ultrasound wavelength. For now, we do not have an answer why the interior of FGs with such small solid inclusions is nevertheless reliably visualized by ultrasound. 

The intensity of the reflected echo signal from the ferrogel layers located beneath the surface not more than 1 mm apart clearly depended on the concentration of iron oxide MPs. The dependence was non-linear (see Figure 10) and was observed in both series of FGs independently of the nature of polysaccharide. The standard deviation of the echo signal increased simultaneously with its brightness (see Table 1 and Table 2). Most likely, it stemmed from the structural inhomogeneity of FGs with high load of solid particles.

The brightness of the echo signal from the boundary also enhanced with iron oxide MPs content like the brightness from the interior (see Figure 9). Meanwhile, unlike the latter, the reflected signal from the boundary linearly depended on the load of MPs. A similar result was obtained in the previous study on ferrogels with embedded iron oxide nanoparticles [40]. According to that work, the reason for the increase of the brightness of the reflected signal was the raise in the stiffness of ferrogels. Good correlation between the brightness and the Young’s modulus was found. In the present study the same correlation was observed. Moreover, the echo signal from the boundary was substantially stronger (2–3 times) than that from the interior of ferrogel. Based on it we may suppose that MPs provided only minor contribution to the signal from the gel/water boundary. In this respect, the echo signal from the boundary rather reflected the mechanical stiffness of the ferrogel surface. Theoretical consideration in support of this conclusion was given earlier in reference [40].

**Figure 12:**
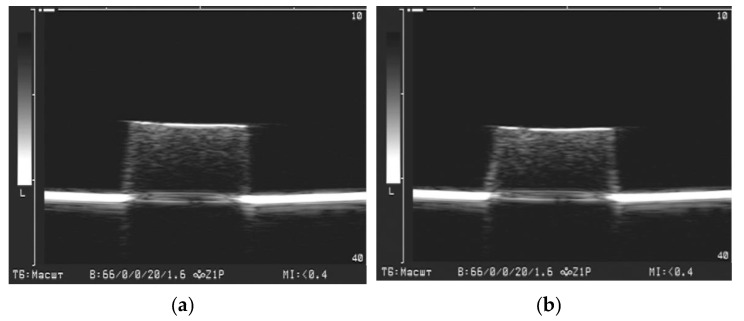
Ultrasound visualization of ferrogel deformation in applied magnetic field. FG with PAAm/Guar network and 23.3% content of iron oxide. (**a**) no field; (**b**) 400 Oe gradient field applied.

As a whole, the obtained results demonstrated the possibilities of medical ultrasound diagnostics for the bioengineering applications, for instance, in tissue engineering and replacement therapy. In particular, ultrasound can be used for the non-invasive evaluation of the shape and dimension of the magnetically driven cell scaffolds and ferrogel implants. Figure 12 illustrates this possibility giving the results of the ultrasound visualization of ferrogel under the action of the applied magnetic field. Ferrogel with PAAm/Guar network with 23.3% of iron oxide MPs is presented. Figure 12a refers to the absence of the field; Figure 12b corresponds to the application of the constant gradient magnetic field 400 Oe. The field was applied by a commercial electromagnet located 5 mm beneath the bottom of ferrogel sample. The details of the experimental setup are given elsewhere [55].

It could be noticed in Figure 12 how the ultrasound imaging visualizes the deformation of the shape and of the dimensions of FG. In this particular case the vertical deformation along the field lines was 6%. In addition to the vertical contraction the parameters of the echo signal brightness changed as well. According to the graphical image analysis, the maximal brightness at the boundary increased from 231 ± 4 up to 239 ± 3 (*n* = 15, *p* > 0.05), while the maximal brightness of the interior increased from 103 ± 2 to 119 ± 4 (*n* = 14, *p* < 0.01). Standard deviation of the signal increased from 13 ± 0.4% up to 17 ± 0.3% (*n* = 15, *p* < 0.01). 

This example shows that during the deformation in the applied magnetic field the brightness of the interior of FG changed more substantially than the brightness at the gel/water boundary. The maximal brightness beneath the boundary and its standard deviation reliably increased under the applied magnetic field. This effect probably stemmed from the structural reorganization of magnetic particles inside FGs. 

Thus, the different effect of the magnetic field on the brightness at the boundary and in the interior likely indicates diverse mechanisms which underlie the acoustic phenomena in the reflection of the ultrasound wave by ferrogels.

Recently [56], the magnetic nanoparticles of iron oxide (magnetite Fe_3_O_4_ from Alfa Aesar, Ward Hill, MA, USA) were used for the detection of their stray magnetic fields by magnetoimpedance sensitive element. MPs were used as a filler in the epoxy resin composite which contained 30% weight fraction of particles. The position of the magnetic inclusion mimicking thrombus in the blood vessel was detected by the measurements of the stray magnetic fields. The combination of two techniques could be an interesting continuation of the present research.

## 5. Conclusions

Biocompatible hydrogels with interpenetrating polymer networks filled with 100–400 nm iron oxide magnetic sub-microparticles in wide range of MPs concentration were synthesized. Physiochemical, magnetic, mechanical, and acoustic properties of FGs were evaluated. All ferrogels demonstrated acoustic reflection from the gel/water boundary and from the FGs interior. The maximum and average brightness at the boundary linearly depended on the FGs stiffness. The echogenicity of the FGs interior was found to depend on the concentration of magnetic particles in FGs network. 

The important conclusion of the present study from the viewpoint of ultrasound visualization is that sub-microparticles show certain advantages in comparison with nanoparticles. Echogenic properties of sub-microparticles allow to obtain information not only on the outer geometry of the gel sample but on its inner structure as well. Ultrasound studies may be used to monitor dynamics of the shape, dimensions, and inner structure of FGs under the application of the external magnetic field. It provides a tool for the optimization of bioengineering devices and gives a means to monitor their properties. It is especially valuable for application of ferrogels in the tissue engineering and regenerative medicine.

In this respect, a certain conclusion might be formulated that the magnetically active implants and scaffolds which are subjected to the external magnetic field to change controllably the dimensions, the shape, and the stiffness should rather be based on magnetic sub-microparticles which allow ultrasound monitoring of their internal arrangements.

## Figures and Tables

**Table 1:** Results of the statistical analysis of the ultrasound visualization for ferrogels with PAAm/Xanthan network filled with different content of iron oxide MPs. The average values with confidence interval at a significance level of *p* = 0.01 (*n* = 60) are present.

Weight Fraction MPs (%)	Brightness (Arbitrary Units)				
	Gel/Water Boundary (Maximum)	Gel/Water Boundary (Average)	Gel Interior (Maximum)	Gel Interior (Average)	Gel Interior (Standard Deviation)
0.0	110 ± 10	100 ± 8	38 ± 4	33 ± 6	1.3 ± 0.1
0.6	126 ± 12	110 ± 10	69 ± 8	40 ± 4	7.2 ± 1.4
1.9	153 ± 12	129 ± 12	82 ± 9	53 ± 6	11.7 ± 1.9
4.3	164 ± 11	135 ± 12	93 ± 9	61 ± 6	12.3 ± 1.2
5.7	177 ± 16	137 ± 13	99 ± 9	65 ± 6	11.8 ± 0.9
8.2	185 ± 11	158 ± 12	100 ± 9	74 ± 5	14.1 ± 2.3
12.9	217 ± 10	167 ± 8	105 ± 7	75 ± 6	14.0 ± 1.2
22.3	265 ± 10	220 ± 12	103 ± 7	70 ± 5	16.0 ± 1.1

**Table 2:** Results of the statistical analysis of the ultrasound visualization for ferrogels with PAAm/Guar network filled with different content of iron oxide MPs. The average values with confidence interval at a significance level of *p* = 0.01 (*n* = 60) are present.

Weight Fraction MPs (%)	Brightness (Arbitrary Units)				
	Gel/Water Boundary (Maximum)	Gel/Water Boundary (Average)	Gel Interior (Maximum)	Gel Interior (Average)	Gel Interior (Standard Deviation)
0.0	110 ± 12	188 ± 14	36 ± 6	33 ± 3	1.2 ± 0.1
0.1	130 ± 12	115 ± 12	65 ± 7	44 ± 5	9.0 ± 1.2
2.9	135 ± 13	105 ± 16	71 ± 6	47 ± 7	6.2 ± 0.5
4.6	160 ± 14	132 ± 12	76 ± 8	52 ± 4	10.1 ± 1.1
10.3	197 ± 14	156 ± 14	95 ± 8	66 ± 7	13.1 ± 0.9
16.0	215 ± 10	177 ± 16	96 ± 10	64 ± 6	13.1 ± 1.3
23.3	255 ± 13	215 ± 12	103 ± 6	74 ± 5	15.0 ± 1.4

## Supplementary Materials

The following are available online at https://www.mdpi.com/article/10.3390/bioengineering8100140/s1, Figure S1: XRD plot of iron oxide sub-micron particles with corresponding Miller indexes, Figure S2: Magnetic hysteresis loop of iron oxide sub-microparticles.
