# Supplementary material for: Echogenic Advantages of Ferrogels Filled with Magnetic Sub-Microparticles
Source: Bioengineering (Basel). 2021 Oct 11;8(10):140. doi: 10.3390/bioengineering8100140 (PMC8533603; doi:10.3390/bioengineering8100140)
Supplement: Supplementary file 1 [file bioengineering-08-00140-s001.zip › bioengineering-1369415-supplementary.pdf]

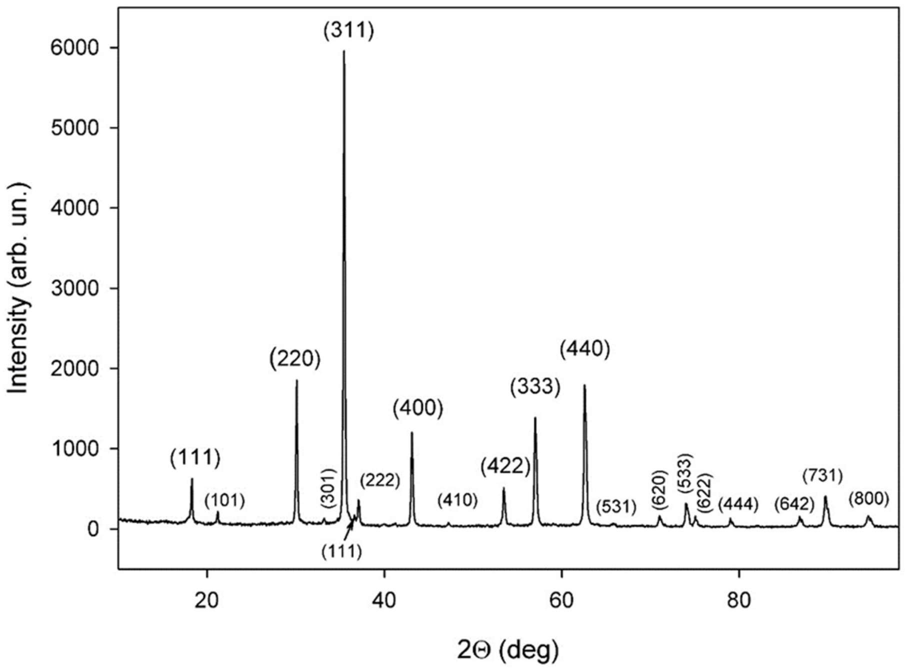

Figure S1. XRD plot of iron oxide sub-micron particles with corresponding Miller indexes.

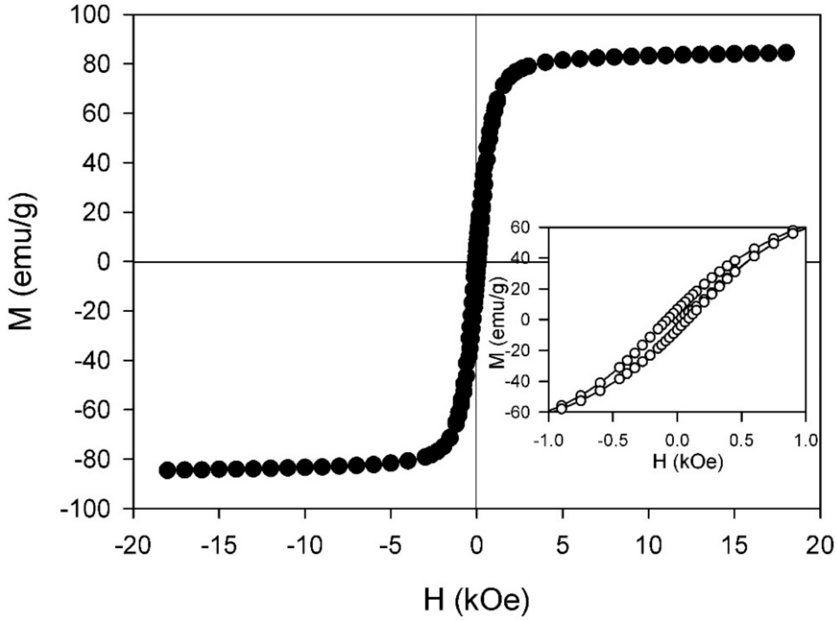

Figure S2. Magnetic hysteresis loop of iron oxide sub-micron particles. The inset shows the part of the loop in low field range.
